# Phylogeography of the Tyrrhenian red deer (*Cervus elaphus corsicanus*) resolved using ancient DNA of radiocarbon-dated subfossils

**DOI:** 10.1038/s41598-017-02359-y

**Published:** 2017-05-24

**Authors:** K. Doan, F. E. Zachos, B. Wilkens, J.-D. Vigne, N. Piotrowska, A. Stanković, B. Jędrzejewska, K. Stefaniak, M. Niedziałkowska

**Affiliations:** 1 College of Inter-Faculty Individual Studies in Mathematics and Natural Sciences, University of Warsaw, Warsaw, Poland; 2 Natural History Museum Vienna, 1010 Vienna, Austria; 3 Department of Nature and Environmental Science, University of Sassari, Sassari, Italy; 4 Muséum National d’Histoire Naturelle - CNRS (InEE) - Sorbonne Universités, Archaeozoology, Archaeobotany, Paris, France; 5 Radiocarbon Laboratory Institute of Physics – Center for Science and Education, Silesian University of Technology, 44-100 Gliwice, Poland; 6 Institute of Genetics and Biotechnology, University of Warsaw, Warsaw, Poland; 7 Institute of Biochemistry and Biophysics Polish Academy of Sciences, 02-106 Warsaw, Poland; 8 The Antiquity of Southeastern Europe Research Centre, University of Warsaw, Warsaw, Poland; 9 Mammal Research Institute Polish Academy of Sciences, 17-230 Białowieża, Poland; 10 Department of Palaeozoology, University of Wrocław, 50-335 Wrocław, Poland

## Abstract

We present ancient mitochondrial DNA analyses of 31 complete cytochrome *b* gene sequences from subfossil red deer remains from the Tyrrhenian islands (Corsica and Sardinia) and mainland Italy in a European-wide phylogeographic framework. Tyrrhenian and North African red deer, both going back to human introductions, were previously the only red deer to harbour the mitochondrial B lineage whose origin, however, remained unknown. Our ancient Italian samples from the central part of the peninsula that were radiocarbon-dated to an age of ca. 6300 to 15 600 cal BP all showed B haplotypes, closely related or even identical to those found on Sardinia. Genetic diversity in the mainland population was considerably higher than on the islands. Together with palaeontological evidence our genetic results identify the Italian Peninsula as the ultimate origin of the B lineage and thus the Tyrrhenian and North African red deer. This is in line with previous biogeographic findings that uncovered distinct intraspecific phylogeographic lineages in Italian mammals, underlining Italy’s status as a hotspot of European mammalian diversity.

## Introduction

Glacial-interglacial cycles, particularly the Last Glacial Maximum (LGM, 26.5 to 19 or 20 ka^1^) have shaped the genetic structure of European temperate mammal species, leaving a strong signature in their genome^1,2^. Unfavourable climatic conditions repeatedly caused the contraction of temperate species’ ranges to glacial refugia in the south of the continent, especially in Iberia, Italy and the Balkans, from which they most recently recolonized their former northern distribution ranges after the LGM. This resulted in conspicuous phylogeographic patterns that can be uncovered through genetic analyses of extant populations (e.g. refs^2,3^).

The red deer (*Cervus elaphus*) is among the best-studied European large mammals, and considerable efforts have been made to reveal its continent-wide genetic structuring. Mitochondrial DNA studies have discovered three distinct phylogeographic haplogroups throughout Europe^4–6^ (for a review see ref.^7^), and data from nuclear microsatellites are in accordance with these findings^8^. The three mtDNA groups comprise (i) a primarily western lineage (A; found from Iberia through western Europe incl. the British Isles to Scandinavia and central Europe); (ii) an eastern lineage (C; ranging from the Balkans north to central and eastern Europe where it co-occurs with the A lineage); and (iii) a geographically restricted and disjunct lineage B that is confined to Sardinia and North Africa, except for single outliers due to human translocation, e.g. on the Isle of Rum in Scotland^9^ and several individuals belonging to this lineages identified in Spain, Bulgaria and Hungary whose origin is not clear^5,10–12^. While lineages A and C can be connected to refugial areas during the LGM in Iberia/southern France (A) and southeastern Europe (C) as also evidenced by the fossil record^13^, lineage B has been somewhat enigmatic. The origin of the Tyrrhenian red deer (*Cervus elaphus corsicanus*) and the North African Barbary red deer (*C*. *e*. *barbarus*), essentially making up haplogroup B, is not entirely clear. The former are indigenous to Sardinia and Corsica. The Corsican population became extinct around 1970 and was re-established with Sardinian animals in the 1980s and 1990s^14^. There seems to be a consensus now that they have been introduced from the Tyrrhenian islands to North Africa or vice versa, but their ultimate origin remains unknown. The Italian mainland is known to harbour divergent genetic lineages in other taxa, including deer (roe deer^15^ and references therein), but extant red deer in Italy are not autochthonous except for a relict population in Mesola in the Po delta area (e.g. ref.^16^). These deer are genetically unique and have recently been described as a distinct subspecies (*C*. *e*. *italicus* ^17^). Their single and exclusive (i.e. private) mtDNA haplotype seems most closely related to the eastern C lineage^18^, although nuclear DNA markers (microsatellites) suggested the Mesola deer to be closest to the Tyrrhenian red deer from Sardinia^8,19,20^. If indeed the island red deer on Corsica and Sardinia are closely related to the Mesola deer, the B lineage might have had a wider distribution in the Italian mainland among the now-extinct indigenous Italian red deer from which founder animals were then translocated to the Tyrrhenian islands and – either from the latter or directly from Italy – to North Africa in historical times. *Cervus elaphus corsicanus* is known from the fossil record of the Tyrrhenian islands since ca. 5000 (Sardinia) and ca 2000 (Corsica) years ago, i.e. red deer first reached the islands long after the LGM sea-level regression during which they perhaps could have colonized them naturally – leaving basically only human introduction as the agent of dispersal^21–25^. This is in accordance with morphological similarities (particularly small body size and simplified antler structure) between Corsican red deer and their indigenous conspecifics from archaeological sites on the Italian mainland^22,25^, although a decrease in body size can also be due to a simple island effect^26^. It has specifically been stated that only ancient DNA (aDNA) analyses of indigenous Italian mainland red deer could settle the question of the origin of the Tyrrhenian red deer^8^. Ancient DNA studies have revealed that the present distribution of phylogeographic lineages may be more limited than before the anthropogenic impacts on large mammal populations, including those of red deer^27^ (for an example in brown bears see refs^28,29^). They have also been performed to elucidate the recolonization of Scandinavia^30^ and specifically to address the colonization of islands: Carden *et al*.^31^ were able to distinguish ancient and modern introductions of red deer to Ireland, and Stanton *et al*.^32^ provided aDNA evidence that red deer populations on Orkney and the Outer Hebrides off Scotland, but not the Inner Hebrides, were probably established through long-distance translocation in the Neolithic.

In the present study we produce ancient mtDNA sequences of red deer from Sardinia, Corsica and mainland Italy and compare them with sequences from extant red deer in a phylogeographic framework to address the following issues: first, we wanted to investigate if lineage B, presently largely confined to Sardinia, Corsica and North Africa, showed a wider distribution in the past, particularly, whether it occurred in extinct indigenous red deer from mainland Italy which would confirm these as the source population for the Tyrrhenian islands and North Africa. And second, since so far the only Corsican red deer that have been analysed genetically^20^ were those reintroduced from Sardinia, we wanted to confirm that the original Corsican population really harboured the same lineage (B) as the red deer on Sardinia. This is not a priori obvious because the two island populations were established at different times in the past (see above).

## Material and Methods

### Sampling

**Figure 1:**
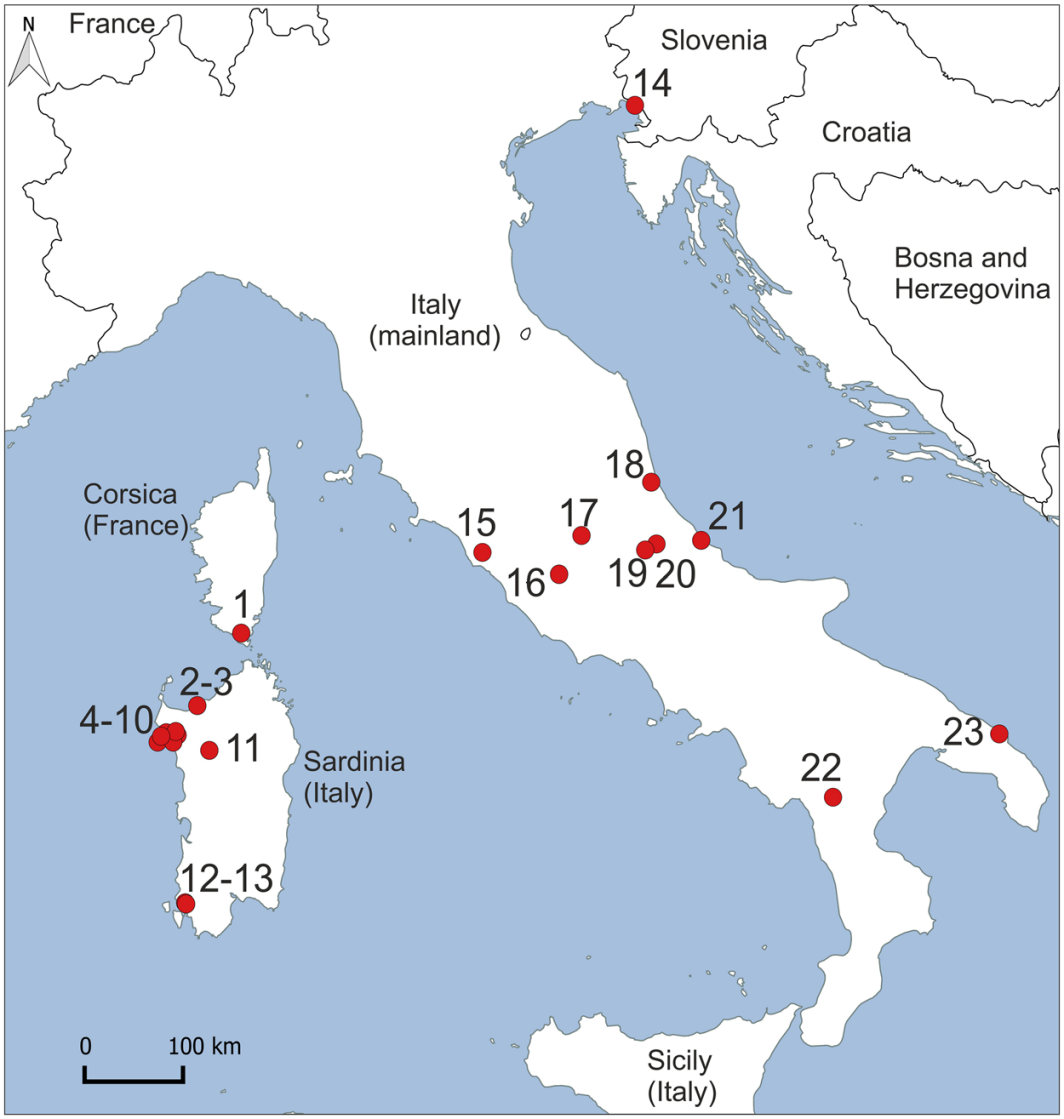
Map with the approximate locations of red deer samples used in this study. 1 – Bufua site, 2 – Sassari, 3 - Village Santa Filitica. 4 - Nuraghe Flumenelongu, Alghero, 5 - Sant’Imbenia village, 6 - Grotta Verde, Alghero, 7 - Nuraghe Talia, Olmedo, 8 - Roman villa nur. Talia, 9 - Necropoli Santu Pedru, 10 - Pozzo sacro La Purissima, 11 - Nuraghe Santu Antine, 12 - Monte Sirai, Sulcis, 13 - Nuraghe Sirai, Sulcis, 14 - Grotta Azzurra di Samatorza TS, 15 - San Pietrino Tolfa, 16 - Grotta Polesini Bagni di Tivoli, 17 - Grotta Continenza Avezzano, 18 – Ripoli, 19 - San Callisto di Popoli, 20 - Grotta dei Piccioni di Bolognano, 21 – Fossacesia, 22 – Latronico, 23 - Punta Le Terrare Brindisi. The map was created with the QGIS software 2.18.3 (http://www.qgis.org/pl/site/) based on a source map from the public Natural Earth website (http://www.naturalearthdata.com/about/).

We analysed 67 samples of red deer remains excavated from archaeological sites in Corsica (France), Sardinia (Italy) and the Italian Peninsula (mainland Italy) (Fig. 1). A complete list of specimens and sample sites (with coordinates and archaeological information) can be found in Table S1.

### DNA extraction, amplification and sequencing

Bone fragments were washed with bleach, rinsed with ddH_2_O, UV-irradiated for 7 min on each side and pulverized in a cryogenic mill (Spex CentriPrep). Approximately 200 mg of bone powder was incubated overnight at 37 °C in 1.6 ml of extraction buffer (0.5 M EDTA, 0.7 mg of proteinase K (20 mg/ml) (Bioline), 0.1 M DTT, 0.5% N-Lauryl sarcosine salt) with constant agitation. After incubation, the supernatant was subjected to phenol:chloroform:isoamyl alcohol (25:24:1, v:v:v) DNA extraction, followed by extraction by chloroform and isopropanol precipitation. The DNA precipitate was resuspended in 60 µl of H_2_O. DNA extraction was performed at the Institute of Genetics and Biotechnology of the University of Warsaw laboratory which is dedicated to ancient DNA analyses.

The whole cytochrome *b* sequence (cytb, 1140 bp) was amplified in multiplex PCRs using twelve overlapping primer pairs described in ref.^33^. Amplifications were performed in a 25 µl reaction volume containing 2 µl mock or ancient DNA extracts, 0.16–0.32 µM forward and reverse primers and 12.5 µl AmpliTaq Gold PCR Master Mix (Applied Biosystem). The amplification conditions were as follows: a 12 min activation step at 95 °C; 30 cycles of 95 °C for 30 s, 53 °C for 30 s, 72 °C for 30 s; and the final extension at 72 °C for 7 min. Multiplex PCR products were used to prepare libraries for sequencing on a Miseq Illumina platform following the protocol from Meyer and Kircher^34^. DNA concentration in each library was determined by real-time PCR using Library Quantification Kits for Illumina platforms (Kapa Biosystems). Libraries were pooled in equimolar ratios and sequenced on a Miseq Illumina platform using MiSeq Reagent Kit v3 at the Institute of Biochemistry and Biophysics, PAS, DNA Research Center Ltd. and the Institute of Genetics and Biotechnology, UW.

Obtained pair-end Illumina reads were assembled with the pandaseq software. Primer sequences were trimmed with mothur^35^, and final sequences were assembled in contigs using the SeqMan Pro software (DNASTAR, Inc.). To ensure authenticity of the sequence data, each sample was amplified in two independent multiplex PCRs, and contigs from both replicates were used to create consensus sequences according to guidelines proposed by ref.^36^.

Some of the primer pairs showed lower amplification efficiency. In order to obtain the whole cytb sequence for all individuals, additional amplifications were performed. Missing cytb fragments were amplified in singleplex PCRs using the same primer pairs as in the multiplex reactions. Amplifications were performed in 25 µl reaction volume containing 2 µl mock or ancient DNA extracts, 0.2 µM forward and reverse primers and 12.5 µl AmpliTaq Gold PCR Master Mix (Applied Biosystem). Amplification conditions consisted of a 12 min activation step at 95 °C, followed by 45 cycles at 95 °C for 30 s, 53 °C for 30 s, 72 °C for 30 s and the final extension at 72 °C for 7 min. PCR products were purified with ExoI/FastAP (Thermo Scientific) and sequenced in both directions on an ABI PRISM 3730xl DNA Sequencer. For each sample, we obtained at least four sequences from independent amplifications.

### Radiocarbon dating

The age of 22 samples from which we successfully obtained whole cytb sequences was determined by radiocarbon dating. It was performed with the accelerator mass spectrometry method (AMS) at Gliwice Absolute Dating Methods Centre (GADAM). The collagen extraction from bones was performed according to the modified Longin’s protocol^37,38^. The bone samples were cleaned in an ultrasonic bath in demineralized water, then dried and ground in a ball mill. The powdered bone was treated with 0.5 M hydrochloric acid to decompose the mineral fraction. Afterwards the residue was rinsed to neutral pH, acidified and kept in 80 °C for 12 hours in an acidic solution (pH = 3). The obtained supernatant was centrifuged, filtered, put in a glass vial and dried in an oven at 75 °C. The subsample of collagen was subjected to graphite preparation with an AGE-3 system equipped with VarioMicro (Elementar) elemental analyser and automated graphitization unit^39,40^. The ^14^C concentrations in graphite produced from our samples, Oxalic Acid II standards and coal blanks were measured in DirectAMS laboratory, Bothell, USA^41,42^. Radiocarbon dates were calibrated using the OxCal v. 4.2 software^43^ and IntCal13 calibration curve^44^. Hereafter the ages are provided as cal BP, *i*.*e*. calibrated age in years before AD 1950.

### Phylogenetic analyses

Red deer sequences obtained in this study were aligned with 73 previously published cytochrome *b* sequences from modern European, Middle Eastern and African red deer specimens in Mafft v. 7.306^45^ (Table S2). Our final alignment was 1131 bp long. We excluded the first 9 bp from the cytb sequences in order to include one sequence from the Mesola deer^18^. We applied four approaches to phylogenetic reconstruction: neighbor-joining, maximum likelihood and Bayesian tree reconstruction as well as a median-joining network. The neighbor-joining tree was constructed in PAUP*^46^ using the HKY + G model which was chosen as the best-fitting substitution model by jModelTest^47^ using Bayesian and Akaike information criteria. The model was chosen among 44 candidate models, excluding those with invariant sites due to the intraspecific character of the analysed data. Branch support was estimated by means of 1000 bootstrap replicates. The maximum likelihood tree was constructed in PhyML v. 3.1^48^ using the same substitution model as in the NJ approach. Tree topology was estimated using the SPR algorithm, and branch support was estimated by means of the aBayes algorithm. The Bayesian approach was performed in MrBayes v. 3.2.6^49^, using partitioning by first, second and third codon positions with a HKY + G model for each partition. In the analysis, base frequencies, rate matrix and shape parameter were unlinked between partitions. Our data was divided into three partitions by codon positions, and each parameter of the substitution model was estimated separately. We ran four Markov chains in two independent analyses for 10 million generations, sampling every 100 generations. The first 25% of samples were discarded as burn-in. The sequence of the Siberian wapiti – *Cervus elaphus sibiricus* (GenBank: AY044862) – was used as an outgroup in all phylogenetic tree reconstructions.

The median-joining network was constructed in PopART v. 1.7^50^. For its presentation we took into account the haplotypes’ geographic distribution and frequencies.

Haplotype and nucleotide diversities for individuals from haplogroup B were calculated with DnaSPv. 5.10.1^51^. We calculated these indices for the Sardinian/Corsican and the Italian mainland red deer separately. The latter was limited to individuals from the central part of the Italian Peninsula (excluding the northeast where lineage C was found, see below). In addition we calculated the indices for all haplogroup B samples, regardless of their origin, to get overall values for this haplogroup. This value was based on all individuals carrying B haplotypes, ancient and contemporary.

## Results

We successfully extracted DNA from 35 (52%) out of 67 samples (21 from Sardinia, 11 from mainland Italy and three from Corsica). Two samples from Sardinia and two from mainland Italy showed poor DNA quality. We were not able to obtain the products of multiplex PCR from the sample I51 and therefore excluded it from further analysis. For the second sample (I11) we found discordances in sequences from two contigs which could have resulted from post-mortem damages. Since a reliable consensus sequence could not be obtained, this sample was also excluded from phylogenetic analyses. For two other samples we were able to obtain only partial cytb sequences, leaving us with 31 samples for which full cytochrome b sequences (1140 bp) were present (Table S1).

Radiocarbon dating confirmed the age of the samples previously determined by archaeological context. The age of the 15 dated Sardinian samples was from 2700 to 550 cal BP. The two Corsican samples were younger (900-350 cal BP), whereas those from the Italian Peninsula (n = 5) were older than those from the two Tyrrhenian islands, ranging from ca. 15 600 to 6300 cal BP (Table S1).

**Figure 2:**
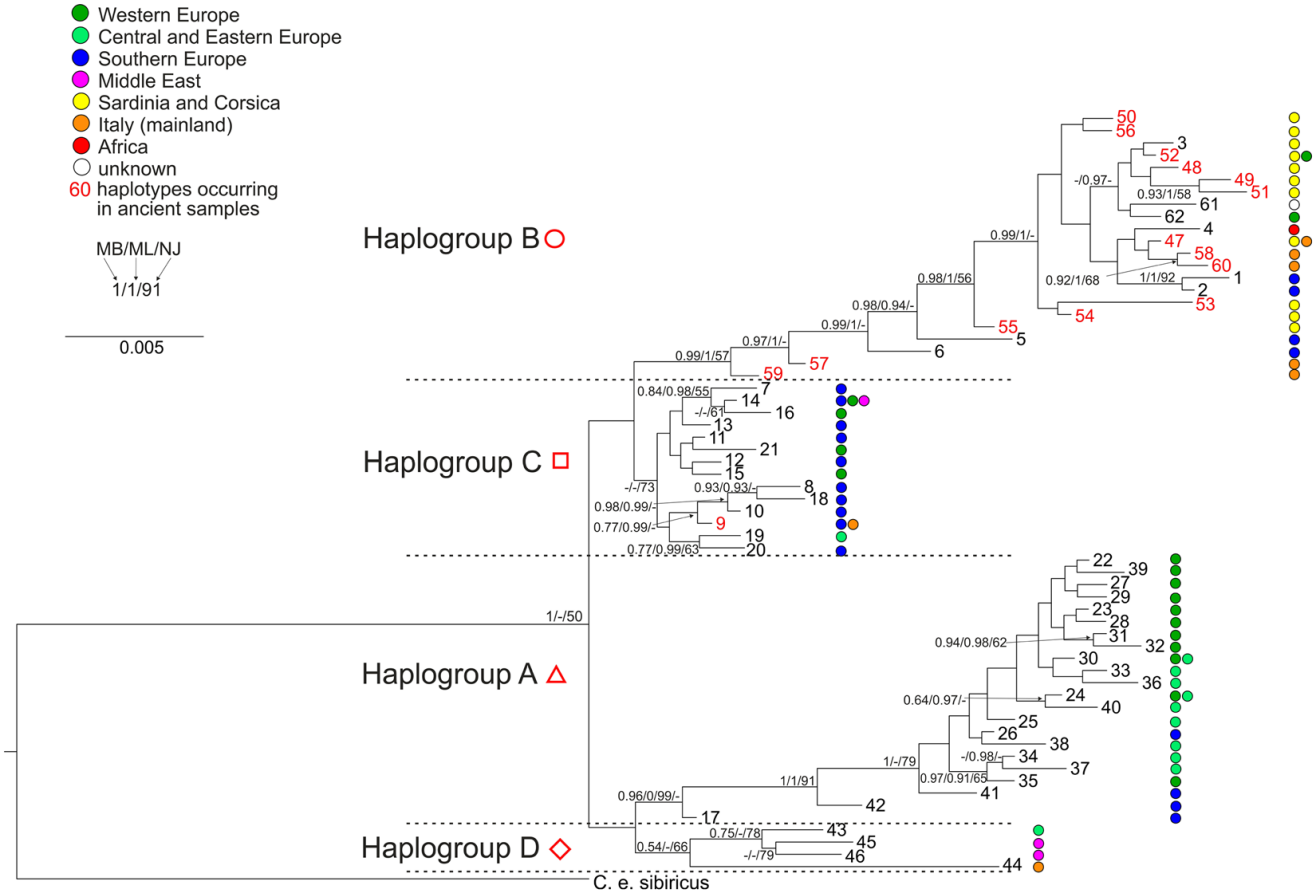
Phylogenetic tree of red deer cytb haplotypes obtained in MrBayes. Coloured circles next to haplotype numbers represent their geographic distribution. Haplotypes occurring in ancient samples are given in red numbers. Numbers at nodes correspond to: Bayesian posterior probability estimated in MrBayes (MB), aBayes support estimated in PhyML (ML) and bootstrap values obtained in PAUP for the neighbor-joining tree (NJ). Node support values lower than 0.5 or 50% are indicated by dashes or not shown.

**Figure 3:**
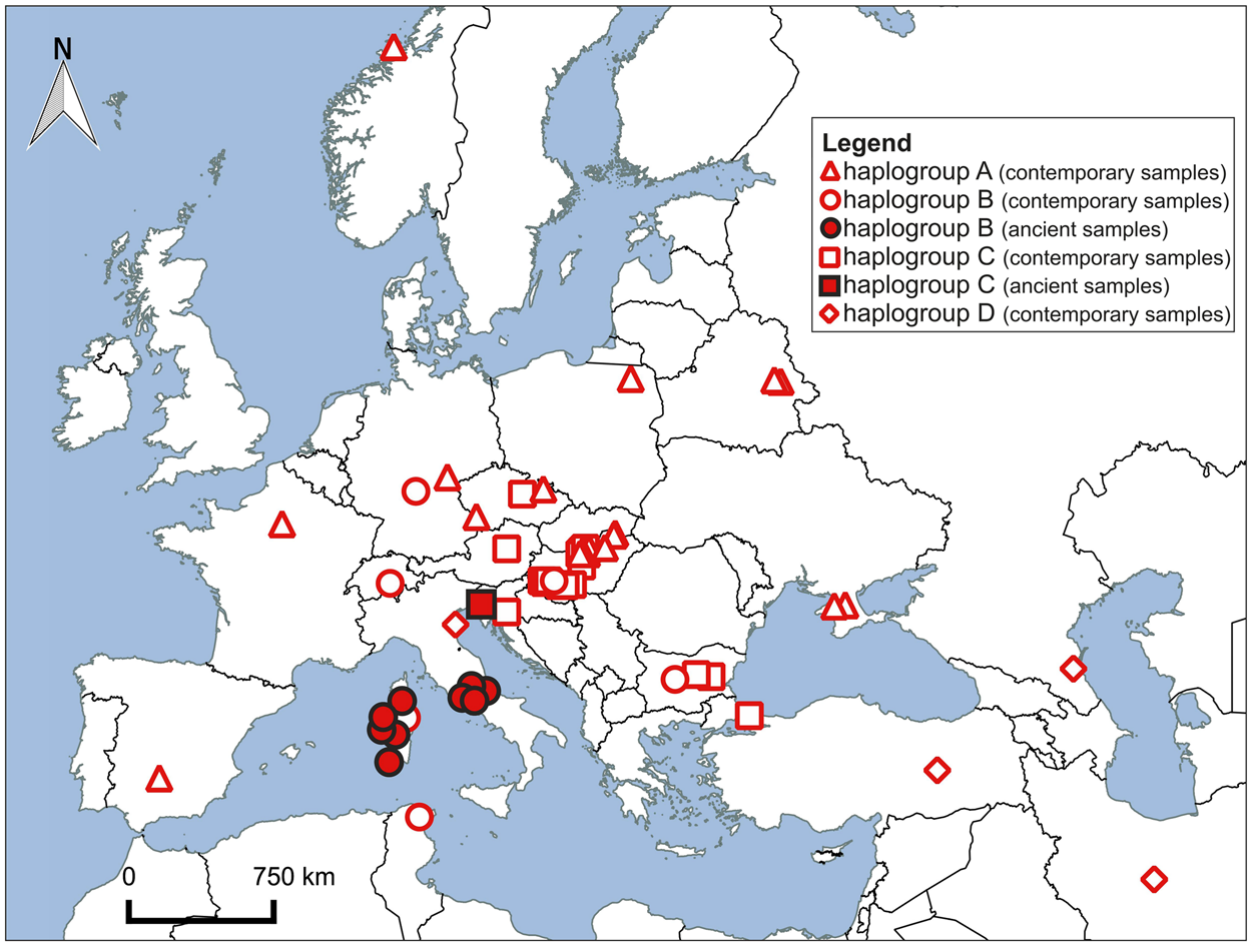
Geographic distribution of mtDNA haplogroups. Haplogroup A is indicated by triangles, haplogroup B by circles, haplogroup C by squares and haplogroup D by diamonds. Red-filled and white-filled signs represent ancient and contemporary samples, respectively. The map was created with the QGIS software 2.18.3 (http://www.qgis.org/pl/site/) based on a source map from the public Natural Earth website (http://www.naturalearthdata.com/about/).

**Figure 4:**
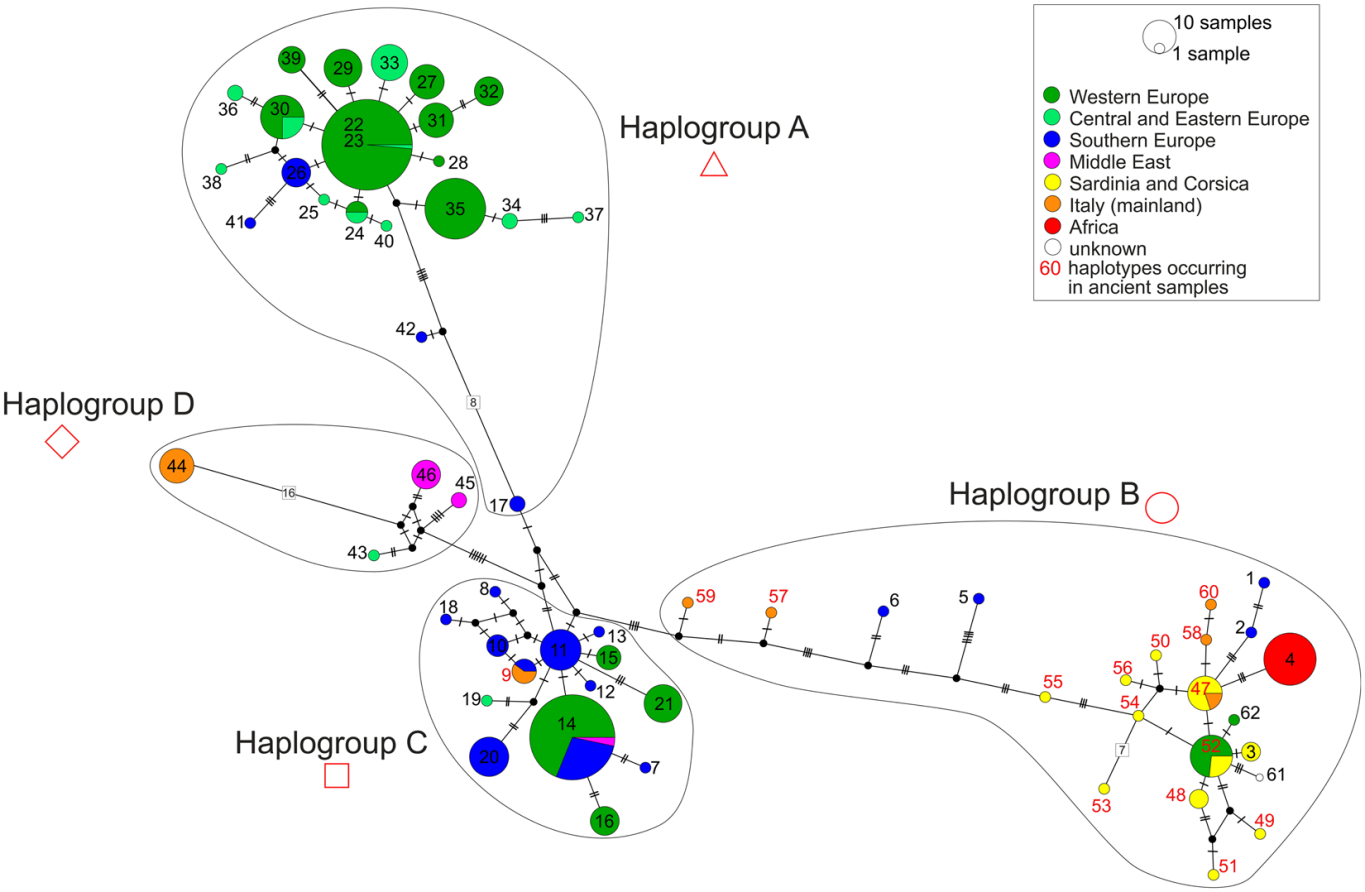
Median-joining haplotype network constructed in PopART. Haplotypes are represented by circles whose sizes are proportional to the number of individuals. Different colours represent geographic distribution. Haplotypes occurring in ancient samples are marked by red numbers. Less than seven mutational steps between haplotypes are indicated by hatch marks; more than seven are indicated by numbers at branches. Missing haplotypes are indicated by small black circles.

The maximum likelihood, neighbor-joining and Bayesian analyses showed similar, well-resolved topologies. The analyses were conducted based on 104 cytb sequences collapsed into 62 haplotypes with an additional outgroup sequence of *C*. *e*. *sibiricus*. As can be seen in the Bayesian phylogenetic tree (Fig. 2), there are four major reciprocally monophyletic lineages. The spatial distribution of these clades is shown in Fig. 3. Most analysed samples from Italy (all except the northern ones) and the Tyrrhenian islands belong to the same clade – haplogroup B (Fig. 3). They cluster together with contemporary B samples from Sardinia, Corsica, Northern Africa, the Swiss Alps, Germany (samples from an enclosure) and Southern European samples from Bulgaria and Hungary (Figs 2 and 4). Among the analysed ancient samples we detected 15 haplotypes, 13 of which were new haplotypes of the haplogroup B (Fig. 4). Combining our data with contemporary sequences available in GenBank allowed us to distinguish a total of 22 known haplotypes in this haplogroup (Figs 2 and 4). One of the most common (H47) occurred both in Sardinia (among samples dated to ca. 2700-1400 cal BP) and in mainland Italy during the Neolithic (Table S1). One haplotype (H48) was shared between Corsica (the sample dated to the 15th–17th century) and Sardinia (samples from the late Bronze Age and the Roman period, Table S1). Haplotype H52 occurred in Sardinian samples dated to ca. 2200-1000 cal BP and contemporary samples from an enclosure in Germany (Tables S1 and S2). The remaining B haplotypes were confined to a single geographic location. One of them (H62) was found in extant red deer from the Swiss Alps, and one was of unknown origin (H61, Table S2). Others were found only on one of the Tyrrhenian islands, on the Italian mainland, in North Africa or Southern Europe (Tables S1 and S2). This is the first confirmation that lineage B occurred in mainland Italy and that the original Corsican population also belonged to this lineage.

Haplotype and nucleotide diversities are given in Table 1. The extinct Italian mainland population from the central part of the peninsula was genetically more diverse than Sardinian and Corsican red deer. In particular, nucleotide diversity was more than three times higher for the mainland compared to the island populations.

**Table 1:** Summary statistics of molecular diversity within haplogroup B of European red deer; n – number of individuals, h – number of haplotypes, Hd – haplotype diversity, π – nucleotide diversity.

Population	n	h	Hd	π
Sardinia and Corsica	25	11	0.87 ± 0.05	0.0024 ± 0.0006
Italian Peninsula	6	5	0.93 ± 0.12	0.0076 ± 0.002
Haplogroup B in total	71	22	0.84 ± 0.03	0.0038 ± 0.0005

Values are based on all available individual cyt b sequences of haplogroup B. For Sardinia and Corsica these were 22 ancient sequences from this study and three contemporary sequences from Ludt *et al*.^4^. The overall number of 71 includes the sequences from this study and all individuals from GenBank. Because in GenBank, often only a single sequence is given per haplotype, we reconstructed individual numbers from the original literature.

Haplotypes H59 and H57, which come from Italian mainland Neolithic and Upper Palaeolithic samples (ca. 6500 and 15600 cal BP, respectively), are consecutive sister groups to the remainder of clade B. Thus, they are less closely related to (and also more divergent from, see Fig. 2) the B haplotypes found in Sardinia, Corsica and North Africa than H1/H2 and H5/H6 found in extant red deer from Bulgaria and Hungary, respectively (Figs 2 and 4, Table S2). H59 and H57 are only few mutational steps away from H11 (haplogroup C, Fig. 4) known from Hungary and Croatia^4,11^. They seem to be intermediate between haplogroups B and C. Haplogroup C is sister to haplogroup B, but this node is only weakly supported (Fig. 2) so that their reciprocal monophyly is not certain. The C lineage mostly comprises deer from Southern and Western Europe (Fig. 3). Three of our ancient Italian samples carried H9 and belonged to this haplogroup (Figs 2 and 4). Contrary to other Italian samples, these came from the northern part of the peninsula, close to the Slovenian border (Fig. 3), where also extant red deer of lineage C occur.

Haplogroup A is well supported and distributed mainly in Western but also in Central, Eastern and even Southern Europe (Fig. 3). H17, the sister group to all other A haplotypes, was found in an extant Hungarian deer. In the network (Fig. 4) it is closer to haplogroup C and in the NJ tree it forms a polytomy together with lineages B and C, albeit with weak support (55%, data not shown). Similar to H57 and H59 it might represent an intermediate haplotype.

Clade D was described in previous studies as the Middle East group^4^, but we decided to consistently use the haplogroup names proposed by Skog *et al*.^5^ Haplogroup D consisted of contemporary deer from Turkey, Iran, the Republic of Dagestan (Russian Federation) in the northern Caucasus – i.e., the distribution range of what is usually called *C*. *elaphus maral* – and, interestingly, the Mesola deer (haplotype 44), the only extant autochthonous Italian population. While it is intriguing that in spite of the Neolithic Italian deer belonging to haplogroup B (and C) the Mesola deer should be more closely related to populations from the Middle East, it has to be emphasized that the support of the Mesola haplotype clustering with the other D haplotypes is rather low and that it is very divergent from them. The network in Fig. 4 shows that it is 16 mutational steps apart from its closest relative, which is a genetic distance similar to that between, for instance, haplogroups A and C, and higher than that separating haplogroups C and D.

## Discussion

We present a phylogeographic analysis of 31 complete cytochrome *b* sequences of ancient red deer specimens from archaeological sites in the Italian Peninsula and on the Tyrrhenian islands. Apart from available data from the relict population of Mesola these are the first DNA sequences from autochthonous Italian red deer and also the first genetic data from the original Corsican red deer population from before its extinction in 1970 and subsequent re-establishment with introduced animals from Sardinia during the 1980s and 1990s.

Ancient Corsican and Sardinian red deer expectedly exhibited mitochondrial haplotypes of lineage B, but the same held true for all ancient red deer from the central Italian mainland, where we even found an identical haplotype to that in an ancient Sardinian red deer. Radiocarbon analysis dated these ancient Italian deer to between 15 600 and 6300 cal BP and thus to a time before long-distance translocations by humans might have blurred natural patterns. This is therefore the first time that the lineage B has been found in unquestionably autochthonous red deer. *C*. *e*. *corsicanus* and *C*. *e*. *barbarus* on the Tyrrhenian islands and in North Africa, respectively, have been introduced there from mainland Italy (directly or indirectly). Moreover all the red deer carrying B haplotypes elsewhere, known from previous studies (British Isles, Spain, Hungary, Bulgaria, Alps, German enclosure), are or at least could be the result of long-distance translocations. The Italian Peninsula, at least its central and perhaps also southern parts, are therefore in all likelihood the natural distribution range of this lineage, a finding that would be in accordance with the presence of a genetically distinct lineage of roe deer in central-southern Italy (*Capreolus capreolus italicus*, see ref.^15^ and references therein) as well as a mtDNA clade endemic to Italy (and also introduced to Sardinia) in wild boar (*Sus scrofa* ^52^, and references therein). The Alps, as a mountain range in a west-east direction, may have acted as a barrier to postglacial recolonization of more northerly parts of Europe (see Hewitt 2000). It cannot be ruled out that the B haplotypes found in Switzerland, Hungary and Bulgaria are relicts of a formerly much wider geographic distribution. However, given the widespread practice of translocating game animals (see e.g. refs^53,54^), an anthropogenic origin of genetic outliers is always a likely explanation in red deer.

Apart from the probable natural distribution of haplogroup B, our results, in yielding 13 new B haplotypes, also indicate that the lineage used to be much more diverse than the present genetic variability in the relict populations of the Tyrrhenian islands and North Africa might suggest. The higher diversity in mainland Italian red deer (5 haplotypes in 6 individuals) compared to that in the island deer (whose nucleotide diversity is more than three times lower) is further evidence, although based on small sample sizes, of the hypothesis that the Italian mainland is the origin of the introduced red deer in Sardinia, Corsica and North Africa (e.g. refs^20,22^). While this seems a plausible conclusion, it has to be conceded that both *C*. *e*. *corsicanus* and *C*. *e*. *barbarus* underwent serious bottlenecks during the 20^th^ century^20,55^ so that their present genetic depletion may be due to anthropogenic impacts in the recent past rather than the consequence of an ancient founder effect. Our 22 ancient Tyrrhenian samples from Sardinia and Corsica yielded seven singleton haplotypes (H49-51 and H53-56 in Fig. 4) that had not previously been found in extant red deer from these islands, confirming a formerly higher genetic diversity in these islands, which is in accordance with a more recent reduction in genetic diversity. The exclusive occurrence of B haplotypes in ancient Tyrrhenian red deer also makes it very likely that the extant Sardinian genetic outlier carrying an A haplotype found by Skog *et al*.^4^ is due to a recent translocation.

The phylogenetic position of the haplotype found in the relict population of Mesola in the Po delta area, the only extant native Italian red deer, is also interesting. Nuclear genetic analyses based on microsatellites have consistently favoured a close relationship of Mesola deer with *C*. *e*. *corsicanus* ^8,20^. This fuelled the hypothesis that the Tyrrhenian red deer were introduced from the Italian mainland. MtDNA-based analyses, on the other hand, have always yielded a somewhat divergent position for the single haplotype present in this genetically depleted population – either intermediate between the western and eastern lineages A and C^5^ or with closer affinities to the eastern lineage^6,18^, including potential affinities with West Asian *C*. *e*. *maral* ^18^ that were also found in the present study. The phylogenetic proximity between Mesola and the C lineage would geographically match the presence of C haplotypes in ancient northern Italian red deer found in the present study, not far away from the site of Mesola (see Fig. 3). The most recent study including Mesola red deer^56^ found them to be highly divergent (as in the present study) and to be closely related to a rare haplotype found in the Polish Carpathians. This “Mesola lineage”, as the authors dubbed it, is in all likelihood identical to our haplogroup D and would thus include the Mesola deer, at least some maternal lineages in Eastern Europe and possibly *C*. *e*. *maral* (although there is a large genetic distance between the latter and the Mesola deer, see Fig. 4). Interestingly, the geographical distribution of *C*. *e*. *maral*, which is now usually thought to comprise Western Asia and Asia Minor only, has been thought to include parts of Eastern Europe as well (see ref.^57^ and references therein). This was recently supported by the interpretation as *C*. *e*. *maral* of subfossil remains from Romania^58^. So, if one is willing to combine Mesola and its Eastern European (maternal) relatives into the same lineage as *C*. *e*. *maral*, the Mesola lineage^56^ would turn out to be identical to what in the first large-scale phylogeographic study of red deer was called the Middle-Eastern lineage^4^ or haplogroup D (present study).

In the absence of the aDNA data presented here, the African-Sardinian lineage could not be placed phylogeographically in the context of the Late Pleistocene and Holocene distribution history of the red deer. Our study has now revealed that it is the refugial lineage of the Italian Peninsula which is known to have harboured red deer during the Last Glacial Maximum^13,59^. However, our study has also confirmed that there were two genetically distinct autochthonous populations of red deer in mainland Italy: one inhabiting the northern and the second occurring in the central and perhaps southern Italian Peninsula as it was hypothesized by the morphometric studies of red deer remains dated to the older Holocene^59^. Like previous studies^27,31,32^ our analyses have shown that aDNA data derived from subfossil material can shed light on otherwise unanswerable questions pertaining to the historical biogeography of red deer (and other taxa), in particular when it comes to the origin of long-established island populations.

## Electronic supplementary material

Supplementary Information
