# Erratum: Phylogeography of the Tyrrhenian red deer (*Cervus elaphus corsicanus*) resolved using ancient DNA of radiocarbon-dated subfossils

**DOI:** 10.1038/s41598-017-12272-z

**Published:** 2017-09-27

**Authors:** K. Doan, F. E. Zachos, B. Wilkens, J.-D. Vigne, N. Piotrowska, A. Stanković, B. Jędrzejewska, K. Stefaniak, M. Niedziałkowska

**Affiliations:** 1 College of Inter-Faculty Individual Studies in Mathematics and Natural Sciences, University of Warsaw, Warsaw, Poland; 2 Natural History Museum Vienna, 1010 Vienna, Austria; 3 Department of Nature and Environmental Science, University of Sassari, Sassari, Italy; 4 Muséum National d’Histoire Naturelle - CNRS (InEE) - Sorbonne Universités, Archaeozoology, Archaeobotany, Paris, France; 5 Radiocarbon Laboratory Institute of Physics – Center for Science and Education, Silesian University of Technology, 44-100 Gliwice, Poland; 6 Institute of Genetics and Biotechnology, University of Warsaw, Warsaw, Poland; 7 Institute of Biochemistry and Biophysics Polish Academy of Sciences, 02-106 Warsaw, Poland; 8 The Antiquity of Southeastern Europe Research Centre, University of Warsaw, Warsaw, Poland; 9 Mammal Research Institute Polish Academy of Sciences, 17-230 Białowieża, Poland; 10 Department of Palaeozoology, University of Wrocław, 50-335 Wrocław, Poland

*Scientific Reports* 7:2331; doi:10.1038/s41598-017-02359-y; Article published online 24 May 2017

This Article contains errors in the Acknowledgements section.

“We thank Maciej Sykut for his help in collecting samples and the preparation of bone material for laboratory analyses. We also thank: Pascal Tramoni (INRAP Méditerranée) for providing samples from Bufua; Sandrine Grouard (MNHN, Paris) for assistance in the osteological identification of the Bufua archaeological material; the Superintendency of Sardinia for the willingness to provide the material for this study and the University of Pisa for providing samples from sites in the Italian Peninsula. The project was financed by the Ministry of Science and Higher Education (grant no. UMO-2013/11/B/NZ8/00888) and the budget of the Mammal Research Institute Polish Academy of Sciences in Białowieża. KD was supported in part by the EU through the European Social Fund, contract number UDA-POKL.04.01.01-00-072/09-00. This paper is dedicated to the late Anna Stanković (1971–2015), a specialist in ancient DNA studies, a great person, our friend and beloved teacher”.

should read:

“We thank Maciej Sykut for his help in collecting samples and the preparation of bone material for laboratory analyses. We also thank: Pascal Tramoni (INRAP Méditerranée) for providing samples from Bufua; Sandrine Grouard (MNHN, Paris) for assistance in the osteological identification of the Bufua archaeological material; the Superintendency of Sardinia for the willingness to provide the material for this study and the University of Pisa for providing samples from sites in the Italian Peninsula. The project was financed by National Science Centre (grant no. UMO-2013/11/B/NZ8/00888) and the budget of the Mammal Research Institute Polish Academy of Sciences in Białowieża. KD was supported in part by the EU through the European Social Fund, contract number UDA-POKL.04.01.01-00-072/09-00. This paper is dedicated to the late Anna Stanković (1971–2015), a specialist in ancient DNA studies, a great person, our friend and beloved teacher”.

