# Supplementary material for: Phylogeography of the Tyrrhenian red deer (Cervus elaphus corsicanus) resolved using ancient DNA of radiocarbon-dated subfossils
Source: Sci Rep. 2017 May 24;7:2331. doi: 10.1038/s41598-017-02359-y (PMC5443832; doi:10.1038/s41598-017-02359-y)
Supplement: Supplementary file 1 — Supplementary Information [file 41598_2017_2359_MOESM1_ESM.pdf]

Phylogeography of the Tyrrhenian red deer (*Cervus elaphus corsicanus*) resolved using ancient DNA of radiocarbon-dated subfossils

Doan K.[1], Zachos F.E.[2*], Wilkens B.[3], Vigne J.-D.[4], Piotrowska N[5]., Stankovic A[6,7], Jędrzejewska B.[8], Stefaniak K.[9], Niedziałkowska M.[8]

1 College of Inter-Faculty Individual Studies in Mathematics and Natural Sciences, University of Warsaw, Poland;

2 Natural History Museum Vienna, 1010 Vienna, Austria

3 Department of Nature and Environmental Science, University of Sassari, Italy

4 Muséum National d'Histoire Naturelle - CNRS (InEE) - Sorbonne Universités, Archaeozoology, Archaeobotany, Paris, France

5 Radiocarbon Laboratory Institute of Physics – Center for Science and Education, Silesian University of Technology, 44-100 Gliwice, Poland

6 Institute of Genetics and Biotechnology, University of Warsaw, Poland / Institute of Biochemistry and Biophysics PAS, 02-106 Warsaw, Poland

8 Mammal Research Institute PAS, 17-230 Białowieża, Poland

9 Department of Palaeozoology, University of Wrocław, 50-335 Wrocław, Poland

* corresponding author: frank.zachos@nhm-wien.ac.at

Table S1. List of red deer samples used in aDNA analyses. Calibrated ages are given in years before present, *i.e.* years before AD 1950.

**Name**	**Fragment**	**Latitude**	**Longitude**	**Site**	**Dating reference no.**	**^{14}C Age BP**	**Cal. age (95.4%)**	**Median cal. age**	**Indirect age**	**DNA yielded**	**Haplotype**	**Haplogroup**	**GenBank accesion no.**
I2	phalanx	39.18	8.49	Monte Sirai, Sulcis, Sardinia	GdA-4401	2478 ± 27	2720 - 2435	2585	Phoenician period	YES	52	B	KY313796
I3	phalanx	40.49	8.77	Nuraghe Santu Antine, Sardinia	-	-	-	-	Late Bronze Age	YES	48	B	KY313797
I4	ulna proximal	40.64	8.26	Nuraghe Flumenelongu, Sardinia	GdA-4394	1595 ± 27	1545 - 1410	1470	Roman Empire	YES	56	B	KY313798
I10	scaphocuboid	40.56	8.17	Grotta Verde, Alghero, Sardinia	GdA-4214	2262 ± 30	2350 - 2160	2240	Middle-Late Neolithic	YES	47	B	KY313799
I11	humerus distal	40.87	8.63	Sassari, Sardinia	GdA-4395	584 ± 27	650 – 535	605	Middle Ages	poor quality	-	-	-
I12	1 phalanx	40.64	8.26	Nuraghe Flumenelongu, Sardinia	GdA-4400	1608 ± 29	1555 - 1410	1485	Roman Empire	YES	47	B	KY313800
I14	metatarsus diaphysis	40.56	8.35	Pozzo sacro La Purissima, Sardinia	GdA-4396	1697 ± 27	1695 - 1540	1600	Roman Empire	YES	47	B	KY313801
I17	left alisphenoid	41.48	9.14	Bufua site (Figari, Corse-du-Sud), Corsica	GdA-4219	375 ± 25	500 - 320	450	-	YES	48	B	KY313802
I19	2 phalanx	39.18	8.49	Monte Sirai, Sulcis, Sardinia	GdA-4216	2477 ± 29	2720 - 2380	2585	Phoenician period	YES	49	B	KY313803
I20	metatarsus proximal	40.61	8.21	Sant'Imbenia village, Sardinia	-	-	-	-	Iron Age	YES	47	B	KY313804
I21	metatarsus proximal	40.65	8.38	Roman villa nur.Talia, Sardinia	GdA-4392	2179 ± 27	2310 - 2120	2240	Roman Empire	YES	50	B	KY313805
I22	metatarsus distal	40.64	8.26	Nuraghe Flumenelongu, Sardinia	-	-	-	-	Roman Empire	YES	48	B	KY313806
I25	1 phalanx	40.56	8.35	Pozzo sacro La Purissima, Sardinia	GdA-4217	1627 ± 27	1600 - 1415	1530	Roman Empire	YES	47	B	KY313807
I27	femur proximal	40.61	8.21	Sant'Imbenia village, Sardinia	GdA-4399	2204 ± 28	2320 - 2150	2235	Iron Age	YES	47	B	KY313808
I28	diaphyses fragment of left tibia	41.48	9.14	Bufua site (Figari, Corse-du-Sud), Corsica	GdA-4220	918 ± 25	920 – 770	855	-	YES	51	B	KY313809
I31	calcaneus	39.18	8.49	Monte Sirai, Sulcis, Sardinia	GdA-4403	2419 ± 28	2695 - 2350	2440	Phoenician period	YES	53	B	KY313810
I34	coxale	40.87	8.63	Village Santa Filitica, Sardinia	GdA-4218	1251 ± 26	1275 - 1080	1215	Early Middle Ages	YES	52	B	KY313811
I39	femur	40.65	8.38	Nuraghe Talia,	GdA-4393	2234 ± 28	2335 - 2150	2230	Late Bronze /	YES	52	B	KY313812

				Olmedo, Sardinia					early Iron Age				
I40	phalanx	39.18	8.49	Monte Sirai, Sulcis, Sardinia	-	-	-	-	Phoenician period	YES	52	B	KY313813
I41	1 phalanx	40.56	8.35	Pozzo sacro La Purissima, Sardinia	GdA-4389	1649 ± 27	1620 - 1420	1550	Roman Empire	YES	47	B	KY313814
I42	humerus distal	40.64	8.26	Nuraghe Flumenelongu, Sardinia	-	-	-	-	Roman Empire	YES	54	B	KY313815
I43	proximal extremity of a left metacarpus	41.48	9.14	Bufua site (Figari, Corse-du-Sud), Corsica	-	-	-	-	-	YES	55	B	KY313816
I44	phalanx	40.49	8.77	Nuraghe Santu Antine, Sardinia	GdA-4215	2689 ± 30	2850 - 2750	2790	Late Bronze / early Iron Age	YES	47	B	KY313817
I61	1 phalanx	41.97	12.83	Grotta Polesini Bagni di Tivoli, Italy	-	-	-	-	Epigravettiano (Upper Palaeolithic)	poor quality	-	-	-
I62	2 phalanx	41.97	12.83	Grotta Polesini Bagni di Tivoli, Italy	GdA-4583	13040 ± 45	15820 - 15360	15620	Epigravettiano (Upper Palaeolithic)	YES	57	B	KY313818
I63	2 phalanx	42.29	13.09	Grotta Continenza, Italy	GdA-4584	8515 ± 35	9540 - 9480	9510	Mesolithic	YES	58	B	KY313819
I64	1 phalanx	42.29	13.09	Grotta Continenza, Italy	-	-	-	-	Mesolithic	YES	60	B	KY313820
I66	2 phalanx	45.75	13.71	Grotta Azzurra di Samatorza TS, Italy	GdA-4585	7005 ± 30	7930 - 7760	7850	Mesolithic / Neolithic	YES	9	C	KY313821
I67	3 phalanx	45.75	13.71	Grotta Azzurra di Samatorza TS, Italy	-	-	-	-	Mesolithic / Neolithic	YES	9	C	KY313822
I68	3 phalanx	45.75	13.70	Grotta Azzurra di Samatorza TS, Italy	-	-	-	-	Mesolithic / Neolithic	YES	9	C	KY313823
I69	1 phalanx	42.17	13.83	San Callisto di Popoli, Italy	GdA-4586	5775 ± 25	6 650 - 6 500	6580	Neolithic	YES	59	B	KY313824
I76	1 phalanx	42.22	13.96	Grotta dei Piccioni di Bolognano, Italy	GdA-4587	5470 ± 30	6310 - 6210	6280	Neolithic	YES	47	B	KY313825
I77	2 phalanx	42.22	13.96	Grotta dei Piccioni di Bolognano, Italy	-	-	-	-	Neolithic	YES	47	B	KY313826
I79	1 phalanx	40.63	17.94	Punta Le Terrare Brindisi, Italy	-	-	-	-	Bronze Age	poor quality	-	-	-
I51	phalanx	40.56	8.17	Grotta Verde, Alghero, Sardinia	-	-	-	-	-	poor quality	-	-	-
I1	talus	40.61	8.21	Sant'Imbenia village, Sardinia	-	-	-		Iron Age	NO			

I5	talus	40.62	8.40	Necropoli Santu Pedru, Sardinia	-	-	-	-	Punic period	NO
I6	metatarsus distal	40.49	8.77	Nuraghe Santu Antine, Sardinia	-	-	-	-	Late Bronze Age	NO
I7	calcaneus	39.18	8.49	Monte Sirai, Sulcis, Sardinia	-	-	-	-	Phoenician period	NO
I8	talus	40.62	8.40	Necropoli Santu Pedru, Sardinia	-	-	-	-	Punic period	NO
I9	metatarsus proximal	39.18	8.49	Monte Sirai, Sulcis, Sardinia	-	-	-	-	Phoenician period	NO
I13	tibia distal	39.17	8.50	Nuraghe Sirai, Sulcis, Sardinia	-	-	-	-	Phoenician period	NO
I15	talus	40.62	8.40	Necropoli Santu Pedru, Sardinia	-	-	-	-	Punic period	NO
I16	1 phalanx	40.56	8.35	Pozzo sacro La Purissima, Sardinia	-	-	-	-	Roman Empire	NO
I18	3 phalanx	40.56	8.17	Grotta Verde, Alghero, Sardinia	-	-	-	-	Middle-Late Neolithic	NO
I23	scapula	40.61	8.21	Sant'Imbenia village, Sardinia	-	-	-	-	Iron Age	NO
I24	1 phalanx	40.65	8.38	Nuraghe Talia, Olmedo, Sardinia	-	-	-	-	Late Bronze / early Iron Age	NO
I26	1 phalanx	39.17	8.50	Nuraghe Sirai, Sulcis, Sardinia	-	-	-	-	Phoenician period	NO
I29	calcaneus	39.18	8.49	Monte Sirai, Sulcis, Sardinia	-	-	-	-	Phoenician period	NO
I30	humerus distal	40.62	8.40	Necropoli Santu Pedru, Sardinia	-	-	-	-	Punic period	NO
I32	metacarpus	40.56	8.35	Pozzo sacro La Purissima, Sardinia	-	-	-	-	Roman Empire	NO
I35	metatarsus distal	40.09	16.01	Latronico, southern Italy	-	-	-	-	Mesolithic /Chalcolithic	NO
I38	tibia distal	39.17	8.50	Nuraghe Sirai, Sulcis, Sardinia	-	-	-	-	Phoenician period	NO
I45	2 phalanx	40.56	8.17	Grotta Verde, Alghero, Sardinia	-	-	-	-	Middle-Late Neolithic	NO
I46	2 phalanx	40.62	8.40	Necropoli Santu Pedru, Sardinia	-	-	-	-	Punic period	NO
I47	metacarpus diaphysis	40.65	8.38	Nuraghe Talia, Olmedo, Sardinia	-	-	-	-	Late Bronze / early Iron Age	NO
I48	1 phalanx	39.17	8.50	Nuraghe Sirai, Sulcis, Sardinia	-	-	-	-	Phoenician period	NO
I49	calcaneus	40.65	8.38	Nuraghe Talia, Olmedo, Sardinia	-	-	-	-	Late Bronze / early Iron Age	NO
I50	metatarus distal	40.61	8.21	Sant'Imbenia village, Sardinia	-	-	-	-	Iron Age	NO

I60	1 phalanx	41.97	12.83	Grotta Polesini Bagni di Tivoli, Italy	-	-	-	-	Epigravettiano (Upper Palaeolithic)	NO
I70	1 phalanx	42.17	13.83	San Callisto di Popoli, Italy	-	-	-	-	Neolithic	NO
I71	1 phalanx	42.15	11.94	San Pietrino Tolfa, Italy	-	-	-	-	Early Neolithic	NO
I72	1 phalanx	42.73	13.90	Ripoli, Italy	-	-	-	-	Early Neolithic	NO
I73	2 phalanx	42.73	13.90	Ripoli, Italy	-	-	-	-	Early Neolithic	NO
I74	1 phalanx	42.25	14.48	Fossacesia, Italy	-	-	-	-	Late Neolithic	NO
I75	2 phalanx	42.25	14.48	Fossacesia, Italy	-	-	-	-	Late Neolithic	NO
I78	1 phalanx	40.63	17.94	Punta Le Terrare Brindisi, Italy	-	-	-	-	Bronze Age	NO

Further information on sample sites

Corsica

The Bufua 3 small rock shelter was located in South Corsica, next to the Figari airport (site 1 in Fig. 1). A rescue excavation was conducted by A. Pasquet and P. Tramoni[1] in 1990 and yielded a human burial, several hundreds of pottery sherds and numerous lithics made out of obsidian or quartz. Five layers have been recognized. According to the pottery, they have been attributed (from the bottom to the top): to the Early Neolithic (Cardial, layer 5), to the Middle Neolithic (Basien, layer 4), to the Chalcolithic (Terrinien, layer 3), to the early Iron Age (layer 2) and to historical times (layer 1).

Both the Basien and Iron Age layer provided numerous animal bones in a good state of preservation. Their archaeozoological study was conducted by Sandrine Grouard (Muséum national d'Histoire naturelle) under the supervision of Jean-Denis Vigne (CNRS). As it is usual for the Neolithic deposits in Corsica and Sardinia, the 614 identified specimens from layer 4 were dominated by the endemic small ochotonid *Prolagus sardus* (52%). They also comprised 28% of domestic sheep or goat (Caprini), 15% of pig or wild boar (*Sus scrofa* ssp.), 4% of cattle (*Bos taurus*) and a few bones of dog (*Canis familiaris*) and humans. Red deer was lacking in this layer, confirming that the species had not yet been introduced to Corsica at that time[2,3]. *Prolagus sardus* was absent from the Iron Age fauna (layer 2), due to the drastic reduction of the species at that time just before it got extinct[2]. The 200 identified specimens of layer 2 were dominated by domestic caprines (65%) and suids (21%) with a small proportions of cattle (4,5%), dog (1,5%) and hedgehog (one specimen). Fourteen bones of a small-sized red deer were also found in this layer (7%).

As the earliest evidence for deer in Corsica was dated to Roman times[3], it was necessary to get a direct radiocarbon date on the deer bones in order to check if they were still earlier than Classical Antiquity. A small-sized right deer astragalus (measurements: GLl= 43.6mm; Bd= 27.2 mm) was radiocarbon-dated in 1995: Ly-120/OXA-5361: 425±50 BP [1416-1633] cal AD. This evidenced that at least some of the animal bones of layer 2 in fact came from layer 1 and dated to the Late Middle Ages or Modern Times. The radiocarbon dates that have been obtained from two other red deer bones from Layer 2 in the present study (I17, alisphenoid, GdA-4219: 375±25 BP [1447-1631] cal AD; I28, tibia, GdA-4220: 918±25 BP [1030-1181] cal AD; see Table S1) also indicate contaminations by the Central Middle Age and the early Modern Time deposits into the Iron Age layer.

The three deer bones from Bufua 3 which have provided ancient DNA for the present study are therefore unquestionably representatives of the original Corsican population of *Cervus elaphus corsicanus* which became extinct in 1970 and was then re-established through introductions of Sardinian red deer in the 1980s and 1990s.

The Corsican material used in this study is temporarily located at the Muséum National d'Histoire Naturelle in Paris (more specifically, the laboratory "Archaeozoology, archaeobotany") and will eventually be deposited at the Musée archéologique de Sartène in Corsica.

Sardinia

Sardinian samples came from sites 2-13 (Fig 1) which were dated, based on archaeological context, from the Neolithic to the Middle Ages[4]. The Grotta Verde, the most ancient cave site, is located on the cliff on the northwestern coast near Alghero. The faunal remains in the cave are abundant, although deer remains are scarce[5]. One of the antler fragments found at this site had processing marks.

Also within the territory of Alghero are the Nuragic villages of Flumenelongu, Talia and Sant'Imbenia. Flumenelongu and Talia were built during the Bronze Age and were inhabited until the Roman Empire. At the latter site the remains of an important villa rustica were found. The nuragic village of Sant'Imbenia was a port settlement that must have been of great importance for trade and was frequently visited by Phoenician sailors. At the site Bronze Age and Iron Age levels were excavated, yielding, among other things, fragments of elephant ivory, a shell from northwestern Africa and many red deer remains.

The Nuraghe Santu Antine near Torralba, one of the largest and most important nuraghe of Sardinia, is located in the northern part of the island, far from the coast. The faunal remains that were studied have been attributed to the late Bronze Age and come from one of the smaller towers.

During the Iron Age Sardinia was colonized by the Phoenicians and later by the Punics. Some samples were examined from a site in the northern part, Santu Pedru near Olmedo in the territory of Alghero, but the most important sites, Monte Sirai and Nuraghe Sirai, are in the Sulcis area, in the southeastern part of the island. The former Sulcis site was a town founded by Phoenicians on a hill near the sea, while the latter is a site dated to the Bronze Age which was successively occupied by Phoenicians. The sites are located very close to each other. It is assumed that red deer was an animal of great importance for meat and antler processing at both sites.

For the Roman period, in addition to the already mentioned Nuraghe Flumenelongu and Talia sites, we also analysed samples from the well-sanctuary of the Purissima near Alghero. It is dated to the Bronze Age and was frequented until the Roman Imperial age. A cult linked to water was practiced at the sanctuary and numerous ex-votos representing human feet were recovered. The animal remains found at the site are likely to be connected to sacrificial practices.

The Roman villa of Santa Filitica is located on the northern coast. In the first centuries of the Middle Ages, on the ruins of the villa a village was built exploiting the area's resources. Red deer were hunted both for meat and antler processing. Some combs and buckles made of deer antler, of best craftsmanship, were recovered.

The most recent sample is from the urban excavations in the city of Sassari, but unfortunately its quality is poor. All samples from Sardinia are from faunal remains studied by the Archaeozoological Laboratory of the University of Sassari.

The Sardinian material of this study is kept in Sardinia, at the cultural superintendencies in Alghero and Sassari and at the Museo Archeologico Villa Sulcis in Carbonia.

Mainland Italy

On the Italian Peninsula (sites nos 14-23) it was possible to recover more ancient samples[6]. The Cave Polesini is located near Tivoli in the Lazio region and provided a large number of animal remains dating back to the Upper Palaeolithic (epigravettiano). Red deer bones were very abundant. The fauna from this cave has never been studied in depth.

The grotta Azzurra of Samatorza near Trieste[7] is the only site located in northeastern Italy. The excavations of the University of Pisa revealed Mesolithic and Neolithic levels. The study of the Mesolithic fauna highlighted the presence of many wild species, including red deer.

In central and southern Italy, Latronico and grotta Continenza are two important sites showing continuity of life from the Mesolithic to the Neolithic and beyond. Latronico, in the Basilicata region, is a site consisting of a cave system, very rich in faunal remains. The site lies in a hilly position, in an area rich in forests and waters. Hunting was still of great importance in the Neolithic, and red deer remains are frequent.

Grotta Continenza was a cave overlooking the Fucino lake, now dried up, in the Abruzzo region. In the Mesolithic its inhabitants lived on trout fishing and mammal hunting, while in the Neolithic these activities became less important.

For the Neolithic we have samples from some sites in central Italy: San Pietrino Tolfa in the Lazio region; Ripoli, San Callisto di Popoli, grotta dei Piccioni di Bolognano e Fossacesia in the Abruzzo region. With the exception of a cave (grotta dei Piccioni) all sites were village settlements. Domestic mammals prevail in all these sites, but hunting was still practiced, and red deer remains were found.

The most recent site is the village of Punta Le Terrare near Brindisi in the Puglia region, dating back to the Bronze Age. At this time red deer hunting had regained great importance, perhaps as a result of the introduction of the horse, and the percentages of red deer remains are very high.

The material from the Italian mainland used in this study is temporarily stored in the laboratory of archaeozoology of the University of Pisa and will be sent back to the cultural superintendencies of the regions from which it originates.

Table S2. List of contemporary red deer sequences obtained from GenBank and used in phylogenetic analyses.

Origin	Haplotype	Haplogroup	GenBank accesion no.	Reference
Bulgaria	20	C	AF423195	[8]
Hungary	14	C	AF489279	[8]
Iran	46	D	AF489280	[8]
Spain, La Garganta	39	A	AF489281	[8]
Austria	21	C	AY044857	[8]
Germany, Kreuth	31	A	AY044858	[8]
Poland, Masuria	33	A	AY044860	[8]
Norway, Hitra	23	A	AY070221	[8]
Tunis	4	B	AY070222	[8]
Croatia	11	C	AY070225	[8]
Norway, Hitra	23	A	AY070226	[8]
Turkey, Istambul	14	C	AY118197	[8]
Turkey	45	D	AY118199	[8]
Crimea	36	A	AY148966	[8]
Sardinia	3	B	AY244489	[8]
France	27	A	AY244491	[8]
Germany, enclosure	52	B	AY118198	[8]
Czech Republic	14	C	JF893495	[9]
Czech Republic	23	A	JF893496	[9]
Hungary	11	C	KC181310	[10]
Hungary	14	C	KC181311	[10]
Hungary	17	A	KC181312	[10]
Hungary	14	C	KC181313	[10]
Hungary	13	C	KC181314	[10]
Hungary	11	C	KC181315	[10]
Hungary	12	C	KC181316	[10]
Hungary	11	C	KC181317	[10]
Hungary	26	A	KC181318	[10]
Hungary	17	A	KC181319	[10]
Hungary	42	A	KC181320	[10]
Hungary	6	B	KC181321	[10]
Hungary	26	A	KC181326	[10]
Hungary	26	A	KC181327	[10]

Hungary	26	A	KC181328	10
Hungary	26	A	KC181329	10
Hungary	26	A	KC181330	10
Hungary	26	A	KC181332	10
Hungary	41	A	KC181335	10
Hungary	18	C	KC181336	10
Hungary	8	C	KC181341	10
Hungary	10	C	KC181343	10
Hungary	10	C	KC181344	10
Hungary	10	C	KC181345	10
Hungary	10	C	KC181346	10
Hungary	9	C	KC181347	10
Hungary	9	C	KC181348	10
Hungary	5	B	KC181351	10
Belarus	24	A	KC562167	10
Belarus	24	A	KC562168	10
Ukraine	38	A	KC562172	10
Crimea	37	A	KC562173	10
Belarus	40	A	KC562174	10
Belarus	25	A	KC562175	10
Belarus	22	A	KC562176	10
Russia, Dagestan	43	D	KC562177	10
Ukraine	19	C	KC562178	10
Ukraine	34	A	KC562179	10
Ukraine	34	A	KC562180	10
Bulgaria	7	C	KC562185	10
Bulgaria	2	B	KC562186	10
Bulgaria	1	B	KC562187	10
Czech Republic	30	A	KM410139	11
Czech Republic	15	C	KM410140	11
Czech Republic	23	A	KM410141	11
Czech Republic	32	A	KM410142	11
Czech Republic	35	A	KM410143	11
Czech Republic	16	C	KM410144	11

Czech Republic	29	A	KM410145	[11]
Czech Republic	24	A	KM410146	[11]
Czech Republic	27	A	KM410147	[11]
Italy, Mesola	44	D	KP859325	[12]
Unknown	61	B	JF489133	[13]
Swiss Alps	62	B	AJ000021	[14]

References

1. Pasquet, A. & Tramoni, P. Figari: Bufua. *Bilan Sci. du SRA* **8,** 23–25 (1992).
2. Vigne, J.-D. in *The Holocene history of the European vertebrate fauna Modern aspects of research* (ed. Benecke, N.) 295–322 (Verlag Marie Leihdorf, 1999).
3. Vigne, J.-D. Les mammifères post-glaciaires de Corse. Étude archéozoologique. *Supplément à Gall. Préhistoire* **26,** 1–337 (1988).
4. Wilkens, B. *Archeozoologia. Il Mediterraneo, la Storia, la Sardegna*. (Editrice Democratica Sarda, 2012).
5. Carenti, G., Grassi, E., Masala, S. & Wilkens, B. in *Deer and People* (eds. Baker, K., Carden, R. & Madgwick, R.) 23–33 (Windgather Press, 2014).
6. Wilkens, B. *Archeozoologia. Manuale per lo studio dei resti faunistici dell'area mediterranea*. (Editrice Democratica Sarda, 2012).
7. Meluzzi, C. & Wilkens, B. in *Il Mesolitico sul Carso triestino* 28–36 (Società per la preistoria e la protostoria della regione Friuli - Venezia Giulia, 1984).
8. Ludt, C. J., Schroeder, W., Rottmann, O. & Kuehn, R. Mitochondrial DNA phylogeography of red deer (Cervus elaphus). *Mol. Phylogenet. Evol.* **31,** 1064–1083 (2004).
9. Krojerová-Prokesová, J. *et al.* Dybowski's sika deer (Cervus nippon hortulorum): genetic divergence between natural primorian and introduced Czech populations. *J. Hered.* **104,** 312–26 (2013).
10. Markov, G. G., Kuznetsova, M. V, Danilkin, A. A. & Kholodova, M. V. Genetic diversity of the red deer (Cervus elphus L.) in Hungary revealed by cytochrome b gene. *Acta Zool. Bulg.* **67,** 11–17 (2015).
11. Krojerová-Prokešová, J., Barančeková, M. & Koubek, P. Admixture of Eastern and Western European Red Deer Lineages as a Result of Postglacial Recolonization of the Czech Republic (Central Europe). *J. Hered.* **106,** 375–385 (2015).
12. Lorenzini, R. & Garofalo, L. Insights into the evolutionary history of Cervus (Cervidae, tribe Cervini) based on Bayesian analysis of mitochondrial marker sequences, with first indications for a new species. *J. Zool. Syst. Evol. Res.* **53,** 340–349 (2015).
13. Naidu, A., Fitak, R. R., Munguia-Vega, A., La N I, M. E. & Cu Lv, E. Novel primers for complete mitochondrial cytochrome b gene sequencing in mammals. *Mol. Ecol. Resour.* **12,** 191–196 (2012).
14. Randi, E., Mucci, N., Pierpaoli, M. & Douzery, E. New phylogenetic perspectives on the Cervidae (Artiodactyla) are provided by the mitochondrial cytochrome b gene. *Proc. Biol. Sci.* **265,** 793–801 (1998).
